# Datasets on the variations of  minerals in biofortified cassava ( *Manihot esculenta Crantz)*  as a function of storage root portion,  maturity and environment

**DOI:** 10.12688/f1000research.121494.1

**Published:** 2022-05-11

**Authors:** Emmanuel Oladeji Alamu, Busie Maziya-Dixon, Alfred Dixon

**Affiliations:** 1 Food and Nutrition Sciences Laboratory,, International Institute of Tropical Agriculture, Southern Africa, Research and Administration Hub (SARAH) Campus PO Box 310142, Chelstone,, Lusaka, 10101, Zambia; 2 Food and Nutrition Sciences Laboratory, International Institute of Tropical Agriculture, Ibadan, Oyo, 10101, Nigeria; 3 Partnership for Delivery Unit, International Institute of Tropical Agriculture PMB 5230,, Ibadan,, Oyo State,, 10101, Nigeria

**Keywords:** minerals, biofortified cassava roots, clones, environment, sample preparation method, maturity

## Abstract

**Background:** The accurate measurements of the mineral content of cassava roots are vital from a nutritional perspective. The research datasets were from the study assessing the influence of storage root portion, maturity, and environment on the variations of minerals in biofortified cassava roots.

**Methods:** Twenty-five biofortified clones with three varieties as checks were harvested 12 months after planting from five different environments. Also,  a different thirty-nine (39) biofortified cassava clones from the unlimited yield trials (UYTs) that included five (5) white-fleshed varieties (as control) were harvested at the age of 9 and 12 months after planting. In addition, two different methods of sample preparations were employed, using a cork borer and without a cork borer. The samples’ elemental (minerals) analysis was determined using a standard laboratory method.

**Results and conclusion:** The breeders could use the data in their biofortification cassava programs to know the distribution of minerals in the roots and identify the best promising pipelines. Also, the data could be used by food scientists and nutritionists to understand the parts of the roots with optimum minerals to design their processing protocols and to know those genotypes specific to different environments that could be used for various nutrition intervention programs.

## Introduction

Biofortified cassava roots with yellow flesh colour could be a good source of essential minerals and micronutrients and potential functional food. The different values of minerals composition of cassava roots found in the literature have shown many discrepancies beyond those caused by genetic and environmental factors. However, this could be due to inaccurate laboratory measurements, especially errors due to the sample preparation method employed during the analysis. The documented data could help the analysts and processors choose the best sample preparation method to evaluate the mineral compositions of cassava roots. The accurate measurements of the mineral content of cassava roots are vital from a nutritional perspective, especially during the selection stages of the breeding program and for providing quality information on the mineral intake in sub-Saharan Africa. However, there is a shortage of data on the spatial distribution of biofortified cassava storage roots’ mineral contents. The datasets presented the spatial distribution of minerals in biofortified cassava root genotypes and established the sampling method’s effect and harvesting time on their mineral content.

The datasets provide information on the effect of clone and environment on the minerals in biofortified cassava root. Furthermore, they establish which of the two factors strongly influences the mineral contents. In addition, they provide information on the essential minerals in biofortified cassava roots and show any association between the mineral properties assessed. The information the datasets reveals is the distribution of the minerals and their concentrations within the cassava tuber (head, middle, and tail root portions) would primarily benefit cassava breeders, food scientists and nutritionists. Also, the datasets on the effect of clones and environment could help the cassava breeders and processors identify what cassava variety is suitable for growth in all the environments and contains optimum minerals.

Breeders could use the data in their biofortification cassava programs to investigate the distribution of minerals in the roots and identify the best promising pipelines. The data could also be used by food scientists, nutritionists, and processors to understand the parts of the roots with optimum minerals and understand those genotypes specific to different environments used for various nutrition intervention programs. The data could also contribute to Nigeria’s food composition databases.

## Materials and methods

### Source of genetic materials

The genetic materials used to generate the presented datasets were from two trials. The first one comprised of the 39 biofortified cassava genotypes from the unlimited yield trial (UYT) stage of the breeding program and 5 white-fleshed varieties (as checks), totalling 44 genotypes. They were grown in two sets at the research farm of IITA, Ibadan, Nigeria. Set one had 22 biofortified genotypes with white roots varieties as checks, totalling 25 genotypes, while set two had 17 biofortified genotypes with two varieties with white roots as control (totalling 19 genotypes). The source (parent lines with different quality traits) used for the breeding crosses that could dictate the mineral properties of the genotypes was the main difference between the two sets. ^1^ The second one was a multi-environment field trial. It had 25 biofortified cassava genotypes and three varieties with white roots grown in five environments. The five growth locations were from six different agro-ecological zones of Nigeria, namely Ibadan, Ubiaja, Onne, Mokwa and Zaria. The latitude and longitude for the collected samples/data are as follows: Ibadan 7° 38′ N, 3° 89′ E; Onne 4° 41′ N, 7° 09′ E; Ubiaja 6° 65′ N, 6° 38′ E; Mokwa 9° 28′ N, 5° 05′ E; Zaria 11° 16′ N, 7° 63′ E. ^2^ The cassava clones in these trials were planted during the rainy season of July 2005 and 2006 and were grown without fertilizers or herbicides under rainfed conditions. The harvesting of the storage roots was done in April 2006 (9 months) and July 2006 (12 months) after planting (MAP), respectively, where the two middle rows were harvested per plot. Three plants per genotype were harvested and pooled. Six cassava roots of different sizes (two each of the large, medium, and small) were randomly selected and placed in a labelled polythene bag from the pooled roots per genotype. The sample bags were transferred to the laboratory and processed within 24 hours of harvest.

## Preparation of cassava roots for analysis

### Sampling Method 1

The method described by Maziya-Dixon *et al*. ^3^ was employed for the first sample preparation method. The three non-damaged storage roots of different sizes of 900–2300 g (large), 500 to 899 g (medium), and 200 to 499 g (small) were selected at the laboratory level. The roots were washed thoroughly with potable water to remove dirt and sand particles and air-dried on a clean concrete floor for about 5–10 min under atmospheric conditions. After that, the cleaned roots were peeled using a stainless-steel knife and further washed with deionized water to ensure the samples were free of soil contaminants. Next, the peeled and cleaned storage roots were bored through using a cork borer, size 8. The bored depth ranged from 4.6 to 9.7 cm for the head, 3.8 to 7.1 cm for the middle and 3.6 to 6.4 cm for the tail parts of the roots, and each portion was pooled and homogenized. ^3^ The procedures were repeated for each genotype.

### Sampling Method 2

The second sample preparation method also followed the protocol described by Maziya-Dixon *et al*. ^3^ It involved selecting a new set of the three non-damaged storage roots of different sizes (large, medium, and small) cleaned using potable water and air-dried on a clean concrete floor as indicated for Method 1. The storage roots were peeled manually using a stainless-steel knife and rinsed with deionized water. The peeled, cleaned roots were cut longitudinal into four equal parts from the proximal to distal ends. For each root, the two opposite sections were taken, chopped into small pieces, and mixed thoroughly before subsampling. The procedures were repeated for each genotype.

## Determination of macro- and microelement content

The samples for mineral analysis were carefully sub-sampled from the batch samples from the two described sample preparation methods. After sub-sampling, the samples were placed in an already cleaned petri dish using deionized water and dried in an uncorroded conventional oven (Memmert UN 55, GmbH) at 40°C for three days. The dried samples were transferred into well-labelled mineral-free paper envelopes for analysis. The mineral contents were analyzed according to the validated method using inductively coupled optical emission spectrometry (ICP-OES). ^1^^,^^2^^,^^4^ Specifically, a radial view Spectro Ciros CCD ICP-OES (Spectro Analytical Instruments, Kleve, Germany) model was used. Each of the dried samples were weighed (0.03 g) into 1 mg into 50 ml screw-cap polypropylene tubes, and initiation of sample digestion was achieved using 2 ml of HNO _3_ and 0.5 ml of H _2_O _2_. The digestion was completed at 125°C for 120 min using 72-position DigiPrep digestion blocks (SCP Scientific, Baie D’Urf e, Quebec, Canada). An 18 MΩ.cm of water was used to make the final volume of 25 ml of the sample. The digested sample was injected at the flow rate was 2.0 ml/min, and the total analysis time per sample was approximately 2.5 min. The calibration curves for all elements were constructed using the mixtures of high-purity single-element standard solutions in a 4% (v/v) HNO _3_ matrix. The software algorithms were used for the background correction of all wavelengths and spectral interferences, as described by Wheal *et al*. ^4^

## Dataset description and validation

The samples from the two trials (44 + 28 biofortified clones and check varieties) were run in duplicate at the laboratory level. However, for quality data collection, there was an analysis of a mixture of all elements in 4% HNO _3_ (drift correction solution) at every 25 samples. It helped to account for within-run variation in the flows. Furthermore, the blank subtraction, drift correction, and mass and volume adjustments were made offline. The study used six different reference materials (RMs) for the data quality establishment purchased from the US National Institute of Standards and Technology (NIST, Gaithersburg, MD, USA). ^4^ The macro elements of focus in the data include calcium (Ca), magnesium (Mg), sodium (Na), potassium (K), phosphorus (P), and sulphur (S). At the same time, the microelements were Iron (Fe), Manganese (Mn), Boron (B), Copper (Cu), Molybdenum (Mo), Cobalt (Co), Nickel (Ni), Zinc (Zn) and Aluminium (Al), respectively. They were measured on an mg/kg dry weight basis. The storage portions included the proximal (A), middle (B), distal (C), the average ABC (AV) and longitudinal (L).

## Data availability

### Underlying data

Dryad: Quantitative Assessment of Trace and Macro Element Compositions of Cassava (Manihot esculenta) Storage Roots Enriched with Β-Carotene as Influenced by Genotypes and Growing Locations, https://doi.org/10.5061/dryad.k3j9kd59r. ^5^

This project contains the following underlying data:

• Data_on_the_Variations_of_Macro_and_Microelements_as_influnced_by_sampling_method.xlsx
• Data_on_Trace_and_Macro_Element_in_cassava_roots_as_influenced_by_Genotype_and_environment.csv
• README.csv

Data are available under the terms of the Creative Commons Zero “No rights reserved” data waiver (CC0 1.0 Public domain dedication).
