# Supplementary material for: Datasets on the variations of  minerals in biofortified cassava ( Manihot esculenta Crantz)  as a function of storage root portion,  maturity and environment
Source: F1000Res. 2022 May 11;11:509. [Version 1] doi: 10.12688/f1000research.121494.1 (PMC10090860; doi:10.12688/f1000research.121494.1)
Supplement: Peer review history

# Peer review: Reviewer response for version 1

**DOI:** 10.5256/f1000research.133358.r167845

**Published:** 2023-04-11

**Authors:** Peng Zhang

**Affiliations:** 1 Chinese Academy of Sciences, Shanghai, China

The manuscript reported the building of datasets on the variation of minerals from twenty-five biofortified cassava clones with cassava growth status and environment. Basically, the work is very meaningful and important for guiding cassava breeders and producers for cassava field management. The methods used are suitable and sufficient for the detection of the difference among the parameters. If the authors could provided the data related to the soil nutrient status, including macro and micro-nutrients, it would be more significant.

Are sufficient details of methods and materials provided to allow replication by others?

Yes

Is the rationale for creating the dataset(s) clearly described?

Yes

Are the datasets clearly presented in a useable and accessible format?

Yes

Are the protocols appropriate and is the work technically sound?

Yes

Reviewer Expertise:

Cassava genetic improvement and molecular breeding.

I confirm that I have read this submission and believe that I have an appropriate level of expertise to confirm that it is of an acceptable scientific standard.

# Peer review: Reviewer response for version 1

**DOI:** 10.5256/f1000research.133358.r162494

**Published:** 2023-04-06

**Authors:** Treenut Saithong

**Affiliations:** 1 Center for Agricultural Systems Biology, King Mongkut's University of Technology Thonburi, Bangkok, Bangkok, Thailand

This dataset presented the variation of mineral contents in cassava roots from different genetic varieties and cultivated conditions. The data collection is basic information that could benefit various researches on cassava yield and quality improvement, and also food processing industry. While the data is interesting, I recommended revising the following issues before indexing: 

1. The current version of the manuscript contained various confusing sentences, especially in the Introduction,
  e.g. "In addition, they provide information on the essential minerals in bio-fortified cassava roots and show any association between the mineral properties assessed. The information the datasets reveals is the distribution of the minerals and their concentrations within the cassava tuber (head, middle, and tail root portions) would primarily benefit cassava breeders, food scientists and nutritionists."
  and the first part of Materials and Methods
  e.g. "The source (parent lines with different quality traits) used for the breeding crosses that could dictate the mineral properties of the genotypes was the main difference between the two sets".   
2. It would be useful for later research work, if the source of the size category specified in this study (i.e. "The three non-damaged storage roots of different sizes of 900–2300 g (large), 500 to 899 g (medium), and 200 to 499 g (small) were selected at the laboratory level.") is described.
3. The data set in Excel file was presented in a user-friendly style. However, I found that it might increase the usefulness of the data if the author could also add information on genotype background of trait characteristics. Also, all measured number of mineral content should present with a clear unit.  

Are sufficient details of methods and materials provided to allow replication by others?

Yes

Is the rationale for creating the dataset(s) clearly described?

Yes

Are the datasets clearly presented in a useable and accessible format?

Partly

Are the protocols appropriate and is the work technically sound?

Yes

Reviewer Expertise:

Plant metabolism, systems biology, plant computational modeling

I confirm that I have read this submission and believe that I have an appropriate level of expertise to confirm that it is of an acceptable scientific standard.
